# Accessibility of occupational therapy treatment for at-risk children in low- to middle-income countries: A scoping review

**DOI:** 10.4102/ajod.v14i0.1643

**Published:** 2025-10-15

**Authors:** Lizahn Cloete, Zusange Shweni, Leah-Jade Finnucane, Martine Muller, Christelle van Wyk, Lirié du Plessis

**Affiliations:** 1 Department of Interdisciplinary Health Sciences, Faculty of Medicine and Health Sciences, Stellenbosch University, Stellenbosch, South Africa

**Keywords:** barriers to rehabilitation, accessibility to treatment, occupational therapy service delivery, resource-limited settings, children

## Abstract

**Background:**

Barriers to occupational therapy (OT) treatment in low- to middle-income countries (LMICs) are not well documented, posing challenges for ensuring treatment accessibility.

**Objectives:**

This study focuses on at-risk children aged 0–17 years in LMICs, a vulnerable population facing unique and often overlooked barriers to accessing OT treatment. Given that low-income countries account for 85% of the world’s population, it is imperative to ensure that vulnerable children living in these regions receive adequate attention and resources to support their development and well-being.

**Method:**

This study explored the barriers to the accessibility of OT treatment for at-risk children in LMICs. Following the JBI Manual for Evidence Synthesis – 2024 edition, a search of CINAHL, PubMed, Scopus, PsycINFO and Web of Science yielded eight eligible studies. Records were screened first by title and abstract, and then by full text. All included studies were published within the last 10 years with a focus on at-risk children and/or adolescents who received OT treatment in LMICs.

**Results:**

A shortage of trained professionals is presented as the most common barrier limiting access to OT. Other barriers included limited government funding, lack of resources that impeded the delivery of treatment, social stigma and cultural attitudes, and lack of knowledge and awareness about OT.

**Conclusion:**

Further research is required to explore ways to address these barriers to improve access to OT services.

**Contribution:**

Identified barriers can facilitate actions to increase accessibility to OT interventions for at-risk children in LMICs, with the goal of improved health outcomes and greater social inclusion.

## Introduction

Social barriers, such as poverty, limited access to education, exposure to violence or traumas and neglect, contribute to underperformance in children who are considered at risk (Singh 2024). Ghongkedze (2018) suggest that ‘at-risk’ children face various environmental and structural barriers that put them at a higher risk of experiencing behavioural, developmental or societal problems such as poor academic performance, financial difficulties, neglect and difficulties becoming a positive member of society and problems with socio-emotional skills and coping skills (Goldschmidt & Pedro 2019). For at-risk children, ‘treatment’ refers to the strategies employed by occupational therapy (OT) professionals to meet their occupational needs.

In low- to middle-income countries (LMICs), up to 249 million children under five face poverty, which can negatively impact their physical, social and emotional development (Peters et al. 2020). Furthermore, at-risk children often have limited access to quality education (Maarman & Lamont-Mbawuli 2017) because of the costs of school transport, uniforms and a lack of sensory stimulating toys and textbooks (Nortje 2017). Poor-quality infrastructure, a lack of sanitation and safe running water and a lack of classrooms or qualified teachers hinder learning and future employment opportunities, thus perpetuating a cycle of poverty (Ghongkedze 2018). Globally, almost 85% of children live in LMICs, with approximately 40% of children under five experiencing developmental adversities that are caused by impoverished nurturing and learning environments (Black et al. 2017).

Child neglect and poverty are closely linked and often affect families at the same time (Yordy 2023). Children’s Bureau (2019) defines neglect as happening when a parent or guardian fails to provide a child with basic needs, such as food, clothes, housing, medical care or supervision, to the point where the child’s safety, health or general well-being is put at risk. This includes not meeting the child’s educational needs (Children’s Bureau 2019). However, being unable to provide does not mean that one is unwilling to provide, and for families in LMICs, incapacity is frequently the reason (Yordy 2023). Access to OT treatment for children in under-resourced contexts is complex. For this reason, targeted treatment and support systems are needed to address these complex issues, including OT treatment (Van Vuuren, Okyere & Aldersey 2020).

### Occupational therapy for at-risk children

Occupational therapy for at-risk children encompasses a multifaceted treatment aimed at enhancing various developmental aspects crucial for their well-being and success (Tanner et al. 2020). Children can benefit from OT assistance in developing their fine motor, gross motor, cognitive and sensory processing skills (Guidry 2022). Occupational therapists help children develop these skills by providing them with opportunities to practise acquired skills in a fun and supportive environment (D’Arrigo et al. 2020). Occupational therapists work on enhancing the social skills of at-risk children through developing communication, conflict resolution skills and building positive relationships (Domitrovich et al. 2017). Coping and social skills can assist the children with managing stress, anxiety, anger and other emotions that are often exasperated in the stressful environments (Zohuri & Dalili 2023). Mondi, Giovanelli and Reynolds (2021) explain that an effective method to reduce anti-social behaviours is to enable at-risk children to implement socio-emotional competencies.

Occupational therapists are adept at evaluating the environment to adapt or modify objects or situations. They also mediate between service providers and role-players, and they are advocates for the rights of at-risk children (Chen & Patten 2021). They assist with transition planning, helping at-risk children develop skills necessary for post-school life, including vocational training, job readiness and independent living skills. In addition, occupational therapists help children develop the skills they require to participate in everyday activities, such as playing, dressing and feeding (Clark & Kingsley 2020). Occupational therapists employ various techniques to address occupational performance barriers among children. These techniques encompass a range of interventions aimed at enhancing children’s adaptation to their environment and advocating for at-risk children and their families by collaborating with relevant stakeholders (Beisbier & Cahill 2021). The interventions provide training to educators, parents and caregivers on how to support children’s development to ensure that at-risk children receive the necessary resources and support so that they may thrive (Anarfi, Ofosu-Mensah & Ababio 2018).

The concept of OT, however, does not always align seamlessly with the cultural beliefs and practices prevalent in LMICs. Van Vuuren et al. (2020) argue that OT treatment, often rooted in Western ideologies of health and well-being, may lack cultural sensitivity and appropriateness for diverse communities in LMICs. Van Vuuren et al. (2020) added that implementing OT without considering cultural contexts can lead to community resistance and potentially undermine the effectiveness of interventions, highlighting the importance of cultural awareness and sensitivity in OT treatment in LMICs. In addition, with limited resources and competing healthcare priorities, some argue that prioritising OT may divert attention and resources away from addressing more urgent health issues, such as infectious diseases, malnutrition or maternal and child health (Regalado et al. 2022).

### A lack of resources

Resource scarcity presents a significant barrier for OT and has an impact on the availability of specialised equipment and materials crucial for effective patient assessment and treatment (Van Niekerk et al. 2023). Healthcare facilities may lack access to up-to-date assessment tools, assistive devices or adaptive equipment, thereby hindering the quality of assessments and treatment (Howard et al. 2022). Resource barriers may restrict access to these materials, impacting the range of treatment that can be offered (Van Vuuren et al. 2020). Moreover, even when equipment is available, insufficient government funding and resource constraints can make it difficult to maintain and replace items as they wear out or become outdated, which leads to reduced treatment quality over time (Van Vuuren et al. 2020).

### Shortage of trained professionals

In many LMICs, occupational therapists face barriers ranging from deficient working conditions to low salaries. In Africa, a shortage of health professionals leads to occupational therapists depending on community-based rehabilitation to deliver services in rural areas (Van Vuuren et al. 2020). Consequently, a significant number of occupational therapists opt to migrate to high-income countries in pursuit of better opportunities (Ledgerd & World Federation of Occupational Therapists 2020). This migration can have a devastating impact on healthcare systems in LMICs, as it deprives them of the skilled professionals they need. For instance, within the past 5 years, over 20% of occupational therapists who obtained their degrees in Ghana have emigrated to high-income countries, searching for safety and better working conditions. This has led to a shortage of occupational therapists in Ghana, thus causing difficulty for children with disabilities to access the treatment they need (Adu Gyamfi et al. 2020).

### Poor infrastructure and a lack of transportation

A lack of basic amenities, such as electricity and clean water, can hinder the effectiveness of therapy (Varela et al. 2019). This can lead to delayed or missed appointments, disrupting the continuity of care. In LMICs, poor infrastructure is further exacerbated by shortages of essential medical supplies and medications, which hurts the quality and effectiveness of service delivery (Kruk et al. 2022). In addition, inadequate infrastructure can result in cramped and poorly equipped therapy spaces, making it difficult to conduct effective therapy sessions and assessments (Cho 2023). Furthermore, rural patients may encounter difficulties accessing OT treatment because of limited transportation options (George et al. 2022), long distances and high transportation costs (Chowdhury & Ravi 2022).

### Language barriers

In LMICs, there is often a wide range of languages spoken, which can make it difficult for children and their parents to access healthcare treatments such as OT (Al Shamsi et al. 2020; Rasi 2020). When the treatment is predominantly offered in a single official or foreign language, it risks alienating large segments of the population who do not speak or understand that language (Rasi 2020). Furthermore, language discrepancies may hinder progress and limit intervention effectiveness (Al Shamsi et al. 2020). This linguistic mismatch can lead to misunderstandings, miscommunication and a reluctance to seek OT treatment (Rasi 2020). Inadequate language ability aggravates barriers to accessing healthcare. This results in the underuse of these services and increased dependence on emergency care (Rasi 2020).

### Conclusion

Despite the potential benefits of OT treatment, several barriers persist in LMICs. The practical implication of this research lies in its ability to uncover obstacles that hinder at-risk children’s access to OT treatment, including geographical, financial and cultural barriers. By pinpointing the barriers prevalent in these regions, this study paves the way for more comprehensive investigations into the effectiveness of OT treatment in LMICs. A scoping review can help consolidate existing literature and promote initiatives such as evidence-based practice guidelines and funding for LMIC-specific OT interventions.

### Aim and objectives

The aim of this study was to explore the barriers to the accessibility of OT treatment for at-risk children in LMICs.

Specific objectives were:

- To map the existing literature on the accessibility of OT treatment used for at-risk children in LMICs
- To determine the areas that require additional research
- To summarise the available data

## Methods

The JBI Manual for Evidence Synthesis (2024) guided the presentation of this scoping review. The Population, Content & Context (PCC) framework (Table 1) was used to generate eligibility criteria for this review.

**TABLE 1:** Population, content and context framework used.

Criteria	Description
Population	**At-risk children (0–17) –** children who face various environmental and structural barriers that put them at higher risk of experiencing behavioural, developmental, or societal problems such as poor academic performance, financial difficulties, neglect and difficulties becoming a positive member of society.
Content	**Occupational therapy (OT) treatment** Occupational therapy, occupational therapist, paediatric occupational therapist, OT activities, et cetera.
Context	**Low- to middle-income countries (LMICs) (**countries with low Gross National Income [GNI] per capita, limited access to essential resources like healthcare and education, and vulnerability to various challenges such as conflict, natural hazards, and economic instability). Afghanistan, Bangladesh, Congo, Egypt, Ghana, India, Indonesia, Kenya, Madagascar, Malawi, Mozambican, Pakistan, Southern Africa, low-resourced settings.

### Eligibility criteria

The inclusion and exclusion criteria are presented in Table 2.

**TABLE 2:** Inclusion and exclusion criteria.

Criteria	Inclusion	Exclusion
Relevance	Studies that directly address or contribute to the review question of the scoping review.	-
Timeframe	Only studies published within the last 10 years (2013–2023) were included, as this will ensure that the review is relevant and captures the most up-to-date knowledge on the review topic. By concentrating on recent research, the review can identify and address current knowledge gaps.	-
Language	-	Research studies not published in English.
Geography	The review includes only studies on low- to middle-income countries, e.g. Afghanistan, Egypt, Ghana, India, Mozambique, Pakistan, South Africa, Vietnam, et cetera.	-
Population	The review targeted studies involving children (individuals between the ages of 0–17 years) of any sex.	-
Interventions	The review considered only studies related to occupational therapy (OT) treatment.	-

### Search strategy

The following databases on the Stellenbosch Library website were systematically searched: CINAHL, PubMed, Scopus, PsycINFO and Web of Science.

### Search string examples

- adolescent AND ‘Occupational Therapy’ AND ‘financial barriers’ AND ‘Low- and middle-income countries’
- ‘at-risk children’ AND ‘Occupational treatment’ AND ‘access limitations’ AND ‘Developing Nation’
- ‘disadvantaged children’ AND ‘Occupational Therapy activities’ AND accessibility AND ‘Lower Middle-Income Country’

### Selection of sources of evidence

The research team screened the titles and abstracts of all retrieved articles to exclude those that clearly did not meet the inclusion criteria (Peters et al. 2020). Two reviewers independently and blindly reviewed articles, while a third reviewer peer-reviewed all selected articles. The team discussed conflicts and differing interpretations. The team started with screening when 80% agreement was achieved. Reviews of titles, abstracts and full-text articles followed the same process. This approach minimised bias, allowing for more refined decisions regarding inclusion or exclusion (Peters et al. 2020).

### Data charting

The new edition of the JBI Manual for Evidence Synthesis guided data charting, served as a structured tool for systematically extracting and organising key information from included studies in a scoping review. It provided a clear framework for the researchers to capture essential details related to the study, such as the author, year of publication, country of publication, aim of the study, study design, sample characteristics and findings or results, facilitating the synthesis and analysis of data.

### Data analysis

According to the JBI guidelines, a simple frequency count of concepts, populations, characteristics or other fields of data content is adequate for analysing data in scoping reviews.

### Ethical considerations

Ethical clearance to conduct this study was obtained from the Stellenbosch University Undergraduate Research Ethics Committee on 16 February 2024 (No. U23/11/301).

## Review findings

**FIGURE 1:**
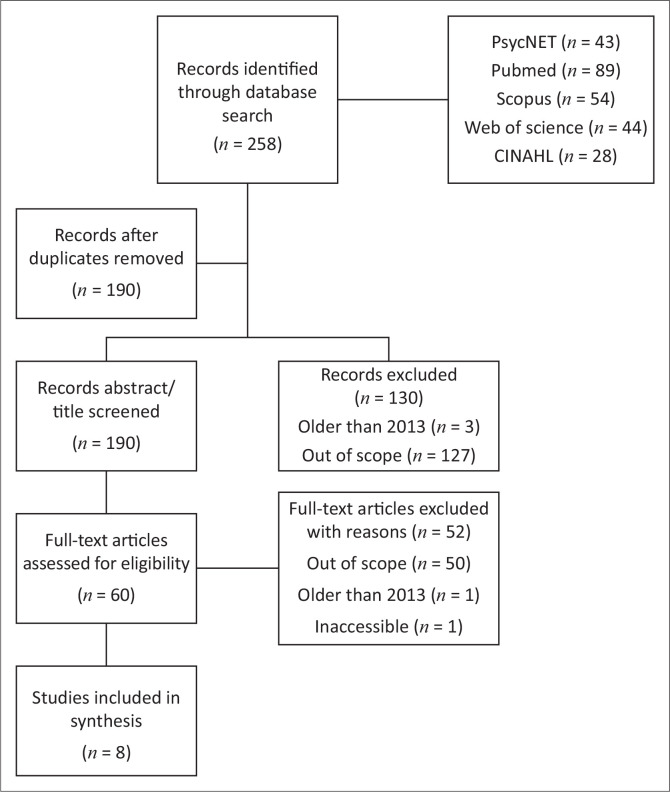
Preferred Reporting Items for Systematic reviews and Meta-Analyses (PRISMA) flowchart illustrating the selection process of articles for inclusion.

In total, 258 records were identified (Figure 1). These searches were distributed across the various databases as follows: PsycNET (43 records), PubMed (89 records), Scopus (54 records), Web of Science (44 records) and CINAHL (28 records).

After removing duplicate records, 190 unique records remained. These 190 records were then screened based on their titles and abstracts. During this screening phase, we excluded 130 records for the following reasons: three records published before 2013, and 127 records were deemed out of scope for OT interventions because of no clear reference to OT. Subsequently, the full texts of the remaining 60 articles were assessed for eligibility.

During this full-text review, 52 articles were excluded for the following reasons: 50 articles were out of scope for OT interventions, one article was older than 2013, and one article was inaccessible. The final review included eight articles (Table 3).

**TABLE 3:** Characteristics of included studies.

Author (year) country	Aim	Methodology	Sample characteristics	Findings/Results
Bunning et al. (2013), Kenya	Determining the type of rehabilitation support for children with disabilities and their families	Qualitative inquiry of rehabilitation services	Health and special educational services (3 health-based rehabilitation services, 2 special schools, 5 units attached to mainstream provision, 28 community-based organisations and local branch of 1 NGO)	There is a need for improved resources for rehabilitation, greater investment in personnel and their training, improved access to the community and better recording systems.
Nota et al. (2015), Zimbabwe	Establishing reasons for the caregiver’s non-compliance with treatment	Qualitative – Descriptive cross-sectional study (Interview administered questionnaire)	Forty caregivers of children (aged 5 years and below) with congenital disabilities & history of defaulting on treatment (39 women, 1 man)	A combination of factors (psychosocial, economic, child-centred and service centred) contributed to caregivers not bringing their children for therapy.
Jindal et al. (2017), India	To explore parents’ (with different socio-economic and cultural backgrounds) perspectives on the rehabilitation of their child with cerebral palsy and their information needs.	Qualitative- Interpretive description approach (semi-structured interviews)	Eighteen caregivers of children with CP (*GMFSC level I to V*)	Parents who focused on the improvement of body structure and function needed education on taking a social view of disability. There is a need for healthcare professionals and services to be more family-centred.
Cloete and Obaigwa (2019), Kenya	To investigate the views of caregivers who have children with autism spectrum disorder in Kenya	Qualitative- descriptive phenomenological study	Twenty-four caregivers of children with ASD	It was a burden to care for a child with a disability. Caregivers and their children experienced isolation and stigmatisation.
Boubour et al. (2020), Malawi	Determining the perceived challenges for paediatric CM survivors.	Qualitative – Exploratory approach (semi-structured in-depth interviews & focus group discussions).	Twenty-three primary caregivers of CM survivors. 11 healthcare workers (in-hospital rehabilitation officers, clinical officers, nurses, and physicians who specialise in the care of CM patients) & 4 community-based rehabilitation workers (based at NGOs or CBOS) who work directly with children in community-based rehabilitation teams.	There is an urgent need to establish further training of rehabilitation personnel at all levels and to build accessible rehabilitation infrastructure.
Yawar and Asif (2022), Pakistan	To establish and put into place a telehealth system in Pakistan to improve the capacity of medical professionals and caregivers of children with developmental disabilities.	Qualitative – Participatory Action Research	Five occupational therapists, one physiotherapist, one speech therapist, one neuro-counsellor.	The application provided access to contextual information for therapy. Knowledge exchange enhanced curricular redesign and advancement in the healthcare system.
Jose et al. (2023), India	To determine the challenges caregivers of children with ASD have when implementing HP.	Qualitative – exploratory survey	Fifty caregivers of children with autism spectrum disorder	A total of 60.5% of caregivers whose children did not receive occupational therapy reported high levels of barriers.
Smith et al. (2018), Uganda	To investigate the accessibility and geographic distribution of school-aged children in Uganda with recognised surgical needs in terms of community-based rehabilitation, assistive devices, familial support, and school reintegration programmes.	Quantitative – cross-sectional study	A total of 1082 children with identified surgical needs, including injuries, acquired deformities, and congenital deformities.	More paediatric surgical services are required to increase community integration. Community-based services were severely lacking for school-aged children in the Northern parts of Uganda.

NGO, non-governmental organisation; ASD, autism spectrum disorders; HP, home programmes; CM, cerebral malaria; ASD, Autism spectrum disorder; HP, home programs; CBOS, Community based organisations; CP, cerebral palsy.

### Description of barriers identified

This section describes the five main barriers identified in accessing OT treatment. The most prominent barrier was the shortage of trained professionals (the most reported barrier and was referred to in six of the eight articles reviewed). Inconsistent training standards compromised the competence of qualified occupational therapists. Uneven distribution of transport, geographic barriers and insufficient governmental funding resulted in inadequate resources for OT services. Four studies reported on cultural attitudes and stigmatisation, which hindered community integration, and only one study reported language barriers. These barriers are presented in Figure 3.

**FIGURE 2:**
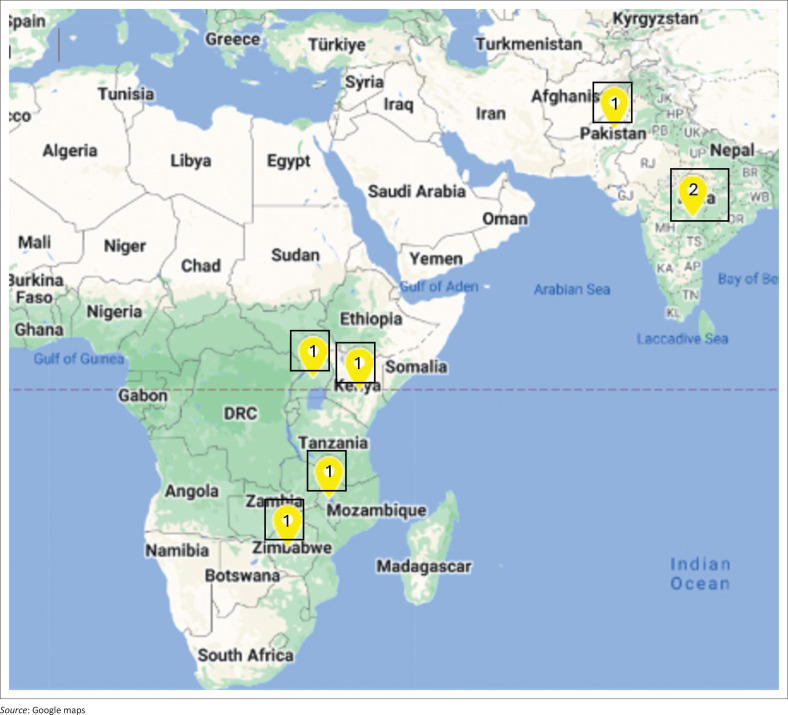
Geographical locations of studies.

**FIGURE 3:**
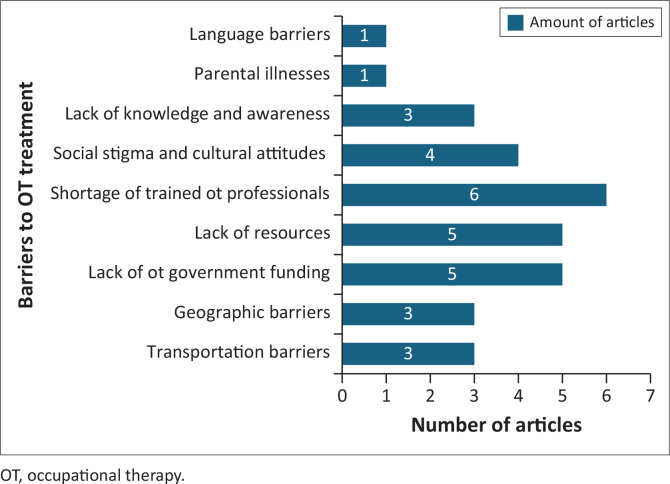
Access barriers to occupational therapy treatment.

### Shortage of trained occupational therapy professionals

Smith et al. (2018) observe that Uganda faces a severe shortage of rehabilitation professionals, with only 30.5% dedicated to work in rehabilitation. Similarly, Cloete and Obaigwa (2019) highlight that Kenya has only 0.2 occupational therapists per 10 000 people. Bunning et al. (2013) explain how there is a lack of learning programmes for rehabilitation professionals in all developing countries, and when they are present, they vary in curricular content and professional competencies. This inconsistency can lead to unqualified professionals addressing both the needs of service users and those relevant to the contexts of rehabilitation provision (Bunning et al. 2013). Jose et al. (2023) observed that Indian healthcare professionals often lacked the necessary appraisal skills and outcome expectancy to implement evidence-based practices effectively. The study also highlighted inadequate support and guidance from rehabilitation professionals; inadequate written instructions and limited consideration of parental input (Jose et al. 2023). Jindal et al. (2017) explain that while children in both India and Canada receive physiotherapy and speech therapy services, there is less exposure to OT treatments, and there are fewer training institutions. Conversely, Canada has an equal number of universities offering physiotherapy and OT courses, leading to more balanced service availability (Jindal et al. 2017). Boubour et al. (2020) describe limited training opportunities in Malawi for rehabilitation specialisation because of funding and opportunity-based barriers, as well as a lack of motivation and leadership. In addition, Yawar and Asif (2022) explain that, in Pakistan, the presence of multiple regional languages poses significant barriers to therapy.

### Transportation and geographic barriers for occupational therapy treatment

Access to OT treatment in Africa is significantly impacted by transportation and geographic barriers. In Uganda, 56.3% of community-based services are concentrated in the Central region, while only 6.0% are in the Northern region, showing a notable disparity in the availability of community-based services (Smith et al. 2018). This uneven distribution severely limits access to essential post-operative care for children in the Northern region (Smith et al. 2018). Bunning et al. (2013) reported that poor public transportation and the urban-centric location of treatments in rural Kenya restrict information dissemination and hinder service uptake by low-income families in rural areas. Nota et al.’s (2015) study found that 35% of the caregivers required resources to set up income-generating projects to help with transport costs. In addition, the study describes how Zimbabwe’s rough terrain complicates wheelchair use for transporting children to hospitals. Approximately two-thirds of the Malawian population live in poverty, with rural families experiencing the highest levels of poverty, poor health outcomes and difficulty accessing healthcare (Boubour et al. 2020). For the Indian population who live in rural settings, long distances to health facilities and unsuitable transportation options exacerbate the barriers to necessary care for children with physical disabilities (Jindal et al. 2017).

### A lack of government funding and resources for occupational therapy services

Uganda’s rehabilitation services rely on unpredictable funding from non-governmental and donor-based agencies, with international development agencies based in Denmark, Norway and the USA providing support (Smith et al. 2018). A lack of knowledge about the benefits of OT treatment in Uganda also affects funding and policy support (Smith et al. 2018). Bunning et al. (2013) reported that Ghana lacked occupational therapists because of insufficient funding and government support. In a smaller study conducted in Kenya, caregivers expressed concerns about the limited access to crucial treatment for children on the autism spectrum, citing little government support, significant financial drain, poor advocacy and inadequate collaborative efforts to implement access to interventions as the key barriers. Cloete and Obaigwa (2019) and Boubour et al. (2020) highlighted that because of inadequate funding for Malawi’s public rehabilitation programmes, most neurorehabilitation infrastructure existed privately through non-governmental organisations (NGOs), making these services costly and unsustainable. Previously, three organisations in Blantyre provided small-scale community-based rehabilitation services, but all were discontinued because of a lack of funding (Boubour et al. 2020). Nota et al. (2015) state that Zimbabwean caregivers experienced financial difficulties and required resources to set up income-generating projects to help them with transport costs. Yawar and Asif (2022) noticed that the scarcity of resources, particularly smartphones and limited Wi-Fi availability, further impedes caregivers’ ability to engage in telehealth sessions and use digital therapy resources effectively. These dual challenges underscore the significant barriers faced by caregivers in accessing therapy remotely (Yawar & Asif 2022). In a qualitative study, Jose et al. (2023) describe a scarcity of necessary resources, such as materials, equipment or tools required to effectively carry out home programmes. Relating to the unavailability of resources for OT treatment after discharge, Smith et al. (2018) suggest that children may require assistive devices, rehabilitation and other specialised social and educational support to address activity barriers and reduce barriers to participation.

### Language barriers

Only one study (conducted in Pakistan) reported on language barriers. Caregivers often speak languages other than Urdu, which therapists may not understand. This language barrier hinders effective communication between therapists and caregivers and has an impact on the quality of care provided. Without adequate language support, caregivers struggle to understand therapy instructions. In turn, this hampers their ability to effectively support their children’s development (Yawar & Asif 2022).

### Cultural attitudes and a lack of knowledge regarding occupational therapy treatment

Bunning et al. (2013) and Cloete and Obaigwa (2019) reported that community members in rural Kenya held beliefs and negative attitudes towards disability that invariably affected services, with cultural superstitions posing barriers to addressing the needs of children with disabilities. Yawar and Asif’s (2022) qualitative study illustrated the stigma that comes with having a child with special needs and/or developmental disabilities in certain cultural contexts. It was found that stigmatisation can deter Pakistani families from seeking therapy or participating in telehealth programmes because of fear of social judgement or discrimination, thus impeding access to essential care for children with developmental disabilities (Yawar & Asif 2022). Jindal et al. (2017) and Nota et al. (2015) reported that Indian and Zimbabwean societies, respectively, presented with negative attitudes towards disability in childhood. Although Zimbabwean caregivers accessed physiotherapy and/or OT, they struggled to distinguish between OT, speech therapy and physiotherapy, often referring to them collectively as exercises (Cloete & Obaigwa 2019; Nota et al. 2015).

## Implications and recommendations

This review explored what has been documented concerning barriers limiting access to OT treatment for at-risk children in LMICs. An alarming finding is the general lack of information about the value of OT treatment for children and their caregivers who are at risk of participating in occupations that facilitate development and community integration. Inadequate numbers of occupational therapists are well-trained to respond to individual and contextual needs.

There is a severe shortage of occupational therapists and rehabilitation professionals in general in African countries (Cloete & Obaigwa 2019; Smith et al. 2018). One contributing factor is the limited availability of OT training in certain countries and the substantial variation in course content and quality, which in turn may lead to inconsistent training standards and lower quality therapy services. (Bunning et al. 2013). Furthermore, support and supervision for therapists are not readily available (Jose et al. 2023), which further impedes access to appropriate services (Bunning et al. 2013).

This scoping review highlights a complex interplay of barriers that affect access to and the quality of OT treatment in Africa and other LMICs. Geographic barriers (Cloete & Obaigwa 2019) and inadequate transportation infrastructure significantly hinders access to OT treatment, particularly in rural areas (Boubour et al. 2020; Nota et al. 2015). Furthermore, poverty restricts access to funds for transport from rural to central areas to attend clinics (Boubour et al. 2020; Nota et al. 2015). Rough terrain, especially in rural areas, further complicates accessibility for wheelchair users or users of other mobility aids (Nota et al. 2015). The concentration of services in urban centres exacerbates the lack of access to treatment, leaving rural populations underserved. For instance, in Uganda, most community-based services are concentrated around the capital Kampala, while rural regions remain largely neglected (Smith et al. 2018). Similar patterns are observed in Kenya, Burkina Faso, Congo, Tanzania, Zimbabwe and Malawi, where poor public transportation and difficult terrain further limit access to essential care.

The sustainability of OT treatments is compromised by a lack of consistent government funding, which leads to a heavy reliance on non-governmental and donor-based agencies (Boubour et al. 2020; Cloete & Obaigwa 2019; Smith et al. 2018). This financial instability affects the availability and quality of treatment across various regions. In Uganda, Kenya, Ghana and Malawi, the dependence on external funding makes OT treatment costly and unsustainable. The lack of government provision restricts the remote provision of therapy because of limited resources and services available to access online services (Yawar & Asif 2022), while a lack of resources at hospitals or clinics restricts the provision of home programmes (Jose et al. 2023). In addition, competing healthcare priorities in countries, such as India, result in limited public support for rehabilitation (Jindal et al. 2017).

Although language barriers were reported in one country, the fact that the language in training schools is limited to dominant languages in countries suggests thatthis problem is widespread. Occupational therapists who are not proficient in indigenous languages will prevent effective communication between therapists and service users (Yawar & Asif 2022). This may not only affect patient progress and healing but also the general understanding and advocacy of OT in African countries. In addition, social stigma and cultural attitudes towards disability deter families from seeking OT treatment, creating barriers to service use. Stigma and discrimination hinder access to necessary care and support for children with disabilities. There is also a pervasive lack of knowledge and awareness about OT treatment among caregivers and communities, with misconceptions about developmental disorders.

### Limitations

Only English articles were utilised, which may have excluded other relevant articles published in different languages. Furthermore, although disability was not a specific search term, it emerged as a prominent limitation in the findings. Occupational therapy is often associated with addressing disabilities, which naturally became a focal point in our research, despite not being the primary intention. The review relied on published articles, which inadvertently may have omitted data from other sources.

## Conclusion

The findings from the research highlight significant barriers to accessing OT treatment for at-risk children in LMICs. The shortage of trained occupational therapists, followed by a lack of resources and inadequate government funding, was identified as the most common barrier limiting access to OT treatment. The barriers identified collectively hinder the delivery of OT treatment, affecting children in rural and underserved regions.
